# Feasibility of tuberculosis drug–induced liver injury screening under programme settings in Southern India

**DOI:** 10.5588/pha.26.0022

**Published:** 2026-08-24

**Authors:** M. Natarajan, M. Verma, K.M. Akshaya, G. Manohar, V. Rathinam, S. Ramalingam, M. Kaleeswari, K. Ganesan, R. Gouroumourty, D. Vasudevan, S. Anandakrishnan, A. Ramachandran, P.M. Ramesh, A. Frederick

**Affiliations:** 1 Department of Respiratory Medicine, Madras Medical College, Chennai, India;; 2 Department of Community and Family Medicine, All India Institute of Medical Sciences, Bathinda, Punjab, India;; 3 Department of Community Medicine, Yenepoya Medical College, Yenepoya (Deemed to be University), Mangaluru, India;; 4 Deputy Director of Health Services, Chennai, Tamil Nadu, India;; 5 Institute of Community Medicine, Madras Medical College, Chennai, India;; 6 Department of Community Medicine, Sri Manakula Vinayagar Medical College and Hospital, Puducherry, India;; 7 Multi-Disciplinary Research Unit, Madras Medical College, Chennai, India;; 8 Department of Respiratory Medicine, Stanley Medical College, Chennai, India;; 9 State Tuberculosis Officer, Chennai, Tamil Nadu, India.

**Keywords:** tuberculosis, M-PHISOR, screening cascade, pharmacovigilance, PwTB, AT-DILI

## Abstract

**BACKGROUND:**

Anti-TB drug–induced liver injury (AT-DILI) is an important treatment-related adverse event. The National TB Elimination Programme (NTEP) recommends baseline liver function testing (LFT), but systematic follow-up monitoring remains fragmented.

**OBJECTIVE:**

To assess the operational feasibility of implementing a screening cascade among persons with TB (PwTB) at high risk for AT-DILI within routine NTEP services.

**METHODS:**

Co-designed implementation research was conducted in three districts of Tamil Nadu between July and December 2025 among adults with drug-sensitive tuberculosis (TB). Persons at high risk were identified using predefined criteria. Feasibility was assessed in implementation terms, focusing on coverage of risk assessment, adherence to the screening cascade, and timeliness of follow-up testing and clinical action.

**RESULTS:**

Of 2,258 PwTB, 52.3% (n = 1,158) were at risk for AT-DILI. Among them, 71.2% (n = 840) underwent baseline LFT testing and 53.9% (n = 637) completed follow-up LFT within 30 days. Eighteen PwTB developed AT-DILI (1.5% incidence under programme conditions) reflecting burden with incomplete follow-up rather than true population burden.

**CONCLUSION:**

Implementation of an AT-DILI screening cascade within routine NTEP services was partially feasible. Risk identification was operationally achievable, but baseline and follow-up testing remained suboptimal. Gaps in follow-up monitoring and documentation highlight the need for strengthened supervision, accountability, and system support before wider scale-up.

Anti-TB drug–induced liver injury (AT-DILI) is a recognised adverse effect of first-line anti-TB therapy (ATT), with reported incidence ranging from 1% to 40% globally.^1^ In high-burden settings such as India, even modest incidence rates translate into a considerable number of persons with TB (PwTB) experiencing hepatotoxicity each year, leading to treatment interruption, morbidity, and mortality.^2^ Vulnerability amplifies due to relatively higher prevalence of coexisting risk factors, such as undernutrition, tobacco, alcohol, HIV co-infection, and other co-morbidities among PwTB.^1,3^ AT-DILI commonly occurs within the first few weeks of initiating the standard ATT.^4,5^ Clinical presentation ranges from asymptomatic derangement of liver enzymes to life-threatening acute liver failure. Symptom-based monitoring can delay the detection and management of AT-DILI. Sequential liver function testing (LFT) identifies asymptomatic patients at risk of developing AT-DILI, enabling timely management.^6^ Despite the Indian National TB Elimination Programme (NTEP) recommending baseline LFTs at treatment initiation, follow-up monitoring is not standardised and is often left to the clinician’s discretion, resulting in inconsistent implementation.^7^ Consequently, early drug safety monitoring remains fragmented. Earlier work from Tamil Nadu under routine programme conditions showed a 5% cumulative incidence of AT-DILI among PwTB initiated on four-drug fixed-dose combination (4FDC) therapy, with most events occurring within the first two weeks of treatment. Notably, some patients remained asymptomatic and were identified only through laboratory testing,^8^ highlighting an opportunity to strengthen early monitoring within existing service delivery mechanisms. Scaling up an intervention requires proactive systems adoption, including integrated risk assessment at treatment initiation, timely laboratory access, standardising documentation, and ensuring a continuum of care for clinical response. Embedding such processes into routine workflows is essential for transitioning from reactive to proactive drug safety monitoring.^9^

A care cascade framework provides a structured method for tracking sequential steps in care, quantifying implementation performance, and identifying operational bottlenecks.^10^ The framework has previously been applied in HIV and other national programmes to support feasibility assessment and programme strengthening.^11,12^ In the present study, feasibility was conceptualised in implementation terms, focusing on coverage of risk assessment, adherence to the screening cascade, and timeliness of follow-up testing and clinical action under routine programme conditions. Against this background, the present study evaluated the operational feasibility of integrating a structured AT-DILI screening and management cascade into routine tuberculosis (TB) services across selected districts. The primary objective was to assess implementation outcomes, including identification and documentation of at-risk patients at treatment initiation, completion of follow-up LFT within the first month, and linkage of detected AT-DILI cases to appropriate clinical management. The secondary objective was to assess service outcomes, namely the incidence of AT-DILI detected within the first month under routine programme conditions, the time from treatment initiation to LFT among at-risk patients, and the time from abnormal LFT to clinical action.

## METHODS

This was a single-arm implementation research exercise using a cohort design and routine programme data from July to December 2025.

### Study settings

Three purposively selected districts of Tamil Nadu (Districts 1, 2, and 3). Tamil Nadu’s NTEP is organised across districts and sub-district administrative TB units, supported by a network of Peripheral Health Institutions. TB units maintain paper registers to record patient notifications, management, and outcomes. Each TB unit is overseen by a Senior TB Treatment Supervisor, who digitises these records into the Ni-*kshay* portal (a case- and web-based electronic TB information management system) using mobile tablets. After the individual is diagnosed with TB at the diagnostic facility, they are referred to the treatment facility, close to the place of residence. For some, the diagnostic and the treatment facility are the same. Till December 2025, Tamil Nadu had operationalised the Tamil Nadu Differentiated TB Care (Tamil Nadu Kaasanoi Erapilla Thittam [TN-KET]) model, a co-designed, context-specific approach aimed at reducing early TB deaths. The model systematically triaged individuals for risk factors related to disease severity at the diagnostic facility and ensures linkage to appropriate levels of care.^13,14^ Districts use Integrated Essential Lab Services (IELSs)-supported labs for convenient follow-up biochemical monitoring.

### Study population and sampling

Adults (≥18 years) with drug-sensitive TB initiated on standard 4FDC therapy were included. We excluded transfers after 2 weeks and pre-existing liver/kidney disease. Universal sampling was adopted.

### Implementation strategy

The intervention was rolled out in three phases: 1) Formative phase: The intervention was informed by prior operational research conducted between September 24 and February 2025, which demonstrated a substantial burden of AT-DILI during the intensive phase and identified locally relevant risk factors.^8^ Findings revealed missed chances for early detection under symptom-only monitoring. Results were shared with State and district TB leaders to co-design a structured screening cascade embedded within NTEP workflows. 2) Implementation phase: Medical officers and programme staffs at the diagnostic facility were sensitised to systematically document predefined AT-DILI risk factors at treatment initiation using the Ni-*kshay* portal and the TN-KET risk stratification framework, where patients are routinely assessed for clinical severity and vulnerability^15^ (triaging) that necessitated inpatient management till stabilisation of the patient; this structure was leveraged to incorporate AT-DILI-specific risk indicators. Patients were classified as high risk if they had at least one predefined criterion (Supplementary Data Table S3). It was the responsibility of the diagnostic facility to record the risk status for AT-DILI in Ni-*kshay* and the differentiated TB care web portal at the time of diagnosis. To facilitate adoption and standardise practice across facilities, simplified cascade algorithms were printed in the local language and displayed to provide point-of-care decision support. Orientation meetings were held with the State TB Officer to standardise screening and referral pathways. Persons at high-risk were advised one follow-up LFT within 15–30 days of treatment initiation. Treatment facilities documented baseline and follow-up LFTs in the TB care portal and referred abnormal cases to district medical colleges for specialist review and regimen adjustment. Monthly reviews within NTEP structures tracked cascade indicators, addressed gaps, and provided feedback. Supportive supervision reinforced adherence to protocol and improved documentation quality. 3) Evaluation phase: We emphasised the completeness and accuracy of documentation within Ni-*kshay*, as the screening cascade relied on routine electronic recording of risk status, laboratory results, and referral actions. During implementation, facilities were encouraged to ensure real-time entry of LFT dates, values, and clinical decisions to strengthen pharmacovigilance tracking within the existing TB information system. Operational feasibility was assessed across the domains of coverage, adherence, and timeliness, while AT-DILI was defined according to the criteria of the Indian Drug-Induced Liver Injury Network^16,17^ (Supplementary Data Table S3).

### Statistical analysis

De-identified data were exported, cleaned, and analysed using SPSS (version 29.0.2.0(20)). Stage-wise cascade proportions were calculated using appropriate denominators at each step (Supplementary Data Table S1). The incidence proportion of AT-DILI within the first month was estimated among persons at high-risk with 95% exact binomial confidence intervals. Time intervals were summarised using medians and interquartile ranges (IQRs). Missing data were not imputed; available case analysis was used, and denominators for each indicator were reported. Cascade indicators were presented overall and stratified by district to assess operational performance across different programmatic contexts. As the objective was a feasibility assessment, formal hypothesis testing was not performed. The AT-DILI screening cascade was presented graphically to illustrate stage-wise completion and drop-offs (Figure).

**FIGURE.**
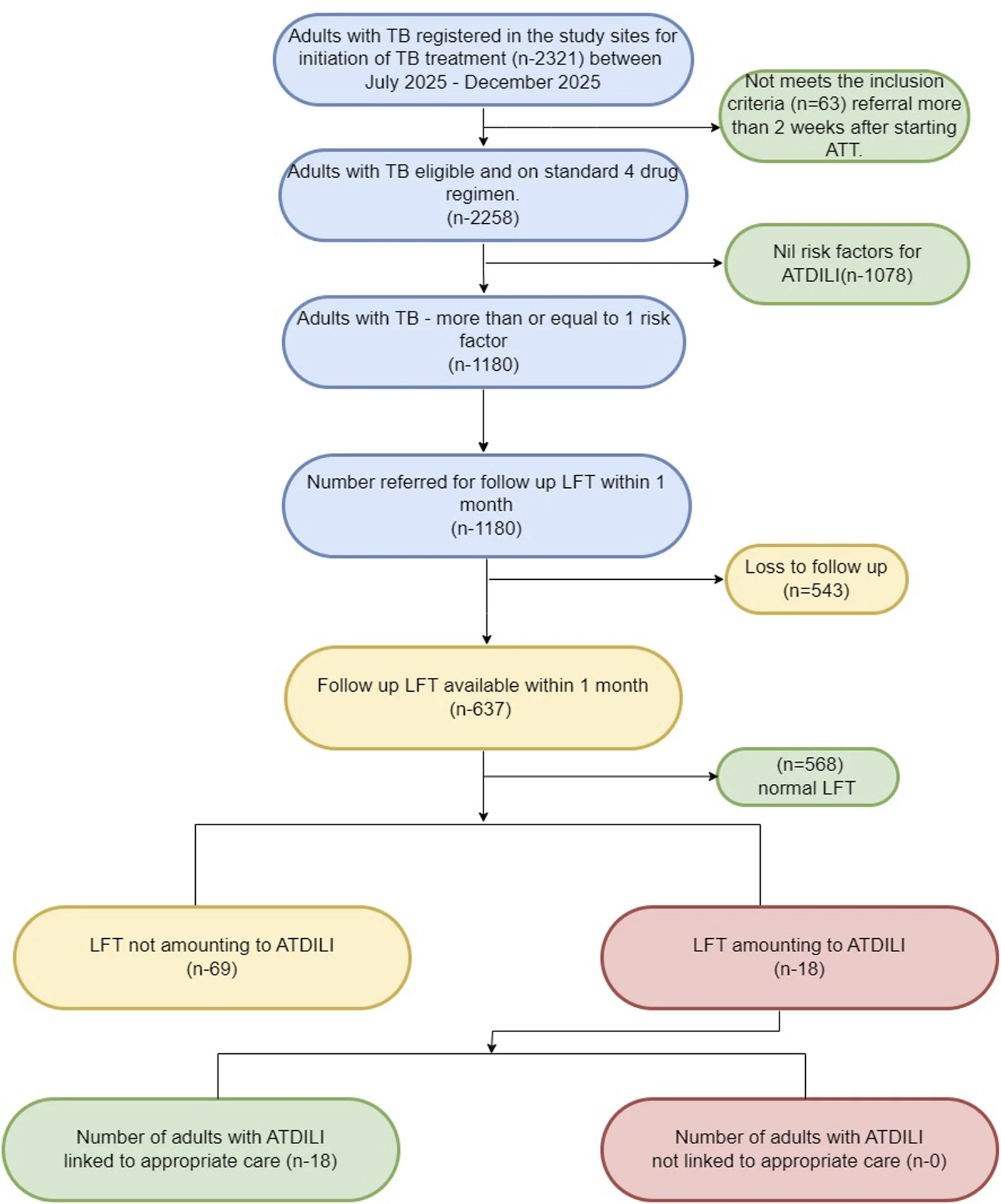
Screening process followed for adults with TB initiated on fixed-dose combination therapy (4FDC-ATT) between July 2025 and December 2025 in three districts of Tamil Nadu, India. TB = tuberculosis; AT-DILI = anti-TB drug–induced liver injury; ATT = anti-TB therapy; LFT = liver function test; 4FDC = four-drug fixed-dose combination.

### Data quality assurance

Study data were extracted from the Ni-*kshay* and Differentiated TB Care web portals. Data completeness was routinely monitored by district and state TB teams. Ten percent of LFT entries were cross-verified against source laboratory records, selected variables were cross-checked against source Ni-*kshay* registers, duplicate entries were removed, and body mass index and TN-KET triage positivity were assessed for concordance across Ni-*kshay* and the TB SeWA portal, where available.

### Ethical statement

Ethics approval was obtained from the ICMR National Institute of Epidemiology (Letter Number NIE/IHEC/A/202506-19; dated 23 June 2025). Informed consent was waived, as de-identified routine programme data were analysed.

## RESULTS

A total of 2,258 adults with TB were registered across three districts: District 1 (n = 459), District 2 (n = 667), and District 3 (n = 1,132). Overall, 69.9% were male. Socio-economic status and disease site were variably documented across districts (Table 1). Among predefined AT-DILI risk factors, 621 patients were aged ≥60 years (27.5%), 137 (6.1%) reported alcohol use, 230 (10.2%) reported tobacco use, and 39 (1.7%) had TB–HIV co-infection. Of all patients, 1,180 (52.3%) had at least one recorded AT-DILI risk factor.

**TABLE 1.** Socio-demographic and clinical characteristics of the adult cohort with tuberculosis (TB) registered on the Ni-*kshay* portal between July 2025 and December 2025, in three districts of Tamil Nadu (N = 2,258).

	Overall, n (%)	District 1, n (%)	District 2, n (%)	District 3, n (%)
Total registered on Ni-*kshay* portal	2,258	459	667	1,132
Gender				
Men	1,579 (69.9)	318 (69.3)	525 (78.7)	736 (65)
Women	679 (30.1)	141 (30.7)	142 (21.3)	396 (35)
Socio-economic status				
Above poverty line	898 (39.8)	302 (65.8)	222 (33.3)	374 (33)
Below poverty line	1,356 (60.2)	153 (33.3)	445 (66.7)	758 (67)
Not recorded	4 (0.1)	4 (0.9)	0	0
Risk factors AT-DILI				
Age ≥60 (Yes)	621 (27.5)	147 (32)	214 (32.1)	260 (23)
Tobacco consumption (Yes)	230 (10.2)	29 (6.3)	170 (25.5)	31 (2.7)
Ever consumed alcohol (Yes)	137 (6.1)	34 (7.4)	25 (3.7)	78 (6.9)
History of diabetes (Yes)	536 (23.7)	82 (17.8)	160 (23.9)	294 (25.9)
Body mass index (Underweight)	383 (17)	101 (22)	103 (15.4)	179 (15.8)
TB–HIV co-infection (Yes)	39 (1.7)	17 (3.7)	7 (1.1)	15 (1.3)
TN-KET triage positive	161 (7.1)	47 (10.2)	65 (9.7)	49 (4.3)
AT-DILI risk factors				
0	1,078 (47.7)	182 (39.7)	229 (34.3)	667 (58.9)
1	778 (34.5)	181 (39.4)	258 (38.7)	339 (29.9)
>1	402 (17.8)	96 (20.9)	180 (27)	126 (11.1)

AT-DILI = anti-TB drug–induced liver injury; TN-KET = Tamil Nadu Kaasanoi Erapilla Thittam.

The district-level screening cascade is depicted in Supplementary Data Table S2. The ATT initiation date was available for 2,252 (99.7%). Risk factors were documented for 2,258 (100%), and 1,180 (52.25) were identified as at risk for AT-DILI (Stage 1). Among those at risk, baseline LFTs were available for 840 (71.2%). Within 30 days of treatment initiation (Stage 2), 637 (53.9%) persons at risk underwent follow-up LFTs, while 551 (46.7%) did not complete follow-up LFT within 30 days, representing the major cascade drop-off. Follow-up completion varied substantially across districts. Among PwTB at risk for AT-DILI, the overall median time to baseline LFT from date of diagnosis was 7 days (IQR: 1–11). The median duration to follow-up LFT was 22 days (IQR: 18–26), ranging from 21 days in District 1 to 23 days in District 3. Overall, only 9 (0.8%) of at-risk individuals completed follow-up LFT within 14 days, and 604 (51.2%) between 15 and 30 days (Table 2). The stage-wise screening cascade is shown in the Figure.

**TABLE 2.** Timeliness of AT-DILI screening among those at risk for AT-DILI among adults with TB started on four fixed-dose combination ATT between July 2025 and December 2025, in three districts of Tamil Nadu (N = 1,180).

Indicators	Overall, n (%)	District 1, n (%)	District 2, n (%)	District 3, n (%)
At risk of AT-DILI	1,180 (100)	277 (100)	438 (100)	465 (100)
Process timeliness				
Time in days from initiation to baseline LFT (Median – IQR)	7 (1.11)	7 (2.11)	6 (1.11)	7 (2.11)
Days from ATT initiation to follow-up LFT (Median – IQR)	22 (18.26)	21 (17.26)	22 (18.26)	22.5 (19.27)
No follow-up done (1)	551 (46.7)	88 (31.8)	36 (8.2)	427 (91.8)
Follow-up LFT completed ≤14 days (2)	9 (0.8)	2 (0.7)	7 (1.6)	0
Follow-up LFT completed 15–30 days (3)	604 (51.2)	181 (65.3)	386 (88.1)	37 (8)
Follow-up LFT not completed within 30 days (4)	16 (1.4)	6 (2.2)	9 (2.1)	1 (0.2)

TB = tuberculosis; AT-DILI = anti-TB drug–induced liver injury; ATT = anti-TB therapy; LFT = liver function test; IQR = interquartile range.

Serum bilirubin, aspartate amino-transferase (AST), and alanine amino-transferase were deranged in 13 (2%), 7 (1.1%), and 50 (7.8%) patients, respectively. Subsequently, all such patients (n = 58, 100%) with abnormal LFTs were referred, and 18 (1.5%) were confirmed to have AT-DILI, yielding an incidence proportion of 1.5% (95% confidence interval: 0.83–2.22) under routine programme conditions.

## DISCUSSION

We conducted implementation research to evaluate the feasibility of embedding a structured AT-DILI screening cascade in NTEP TB care, as assessment in routine settings remains challenging. The key findings suggest that AT-DILI screening was partially feasible. Risk identification at treatment initiation was largely achievable, but completing follow-up LFTs remained the major operational gap. District-level variation further suggests that supervision, documentation, and service readiness influenced cascade performance.

Systematically identifying patients at risk for AT-DILI is operationally achievable. While some districts performed reasonably well, others’ performance was suboptimal, suggesting the need for local system strengthening and supervision. District variations in differentiated TB care and Ni-*kshay* Poshan Yojana highlight that programme performance is not uniform across Tamil Nadu.^18^ Suboptimal outcomes in certain districts may be explained by differences in baseline patient characteristics, notification rates, and the prevalence of risk factors such as HIV, alcohol use, undernutrition, and the relative performance of the IELSs. Strengthening district-specific strategies – through improved infrastructure, targeted community engagement, and streamlined financial disbursement – will be critical to achieving equitable outcomes across the state. The Karnataka (TB–DM screening), tribal Tamil Nadu (sickle cell newborn screening), and multi-state youth club TB screening indicates that it is feasible to implement these integrated screening programmes.^19–21^ The Nepal Provider-Initiated HIV Testing and Counseling study demonstrates that high uptake is achievable when fidelity dimensions are addressed.^22^ Together, these findings highlight that integrating co-morbidity and safety screening into TB programmes requires both operational feasibility and protocol fidelity.

Our preliminary experience shows that baseline testing is feasible but follow-up remains the main bottleneck. The integration relied heavily on the Ni-*kshay* portal, and its role can be enhanced in decision making and follow-ups. In well-performing districts, IELSs operationalised well; hence, seamless follow-up requires strengthening the network. A key challenge was lack of space for entering AST values, essential for diagnosing AT-DILI. Moreover, when baseline LFTs were entered at the diagnostic facility, treatment facilities could not subsequently enter follow-up LFT values. To tackle this, treatment facilities were tasked with entering both baseline and follow-up LFT results. Intermittent non-functionality of the Ni-*kshay* and differentiated TB care portals also contributed to delays and gaps in documentation. Although a formal qualitative assessment was not done, a few practical issues were noted during implementation. Access to LFTs was easier at district hospitals, whereas patients at peripheral centres relied on sample transport, which may have affected follow-up; indirect costs such as travel, wage loss, and the need for an attendant likely added to this. High patient load, competing priorities, and issues such as connectivity and variable familiarity with the mobile application may also have influenced consistent use. So, the barriers were not only patient-related but also health-system-related, particularly laboratory access at peripheral facilities, workflow dependence on sample transport, workforce constraints, and limitations in digital recording. These factors need to be addressed for scale-up beyond Tamil Nadu, including strengthening laboratory networks, sample transport systems, and frontline capacity in resource-constrained settings. This would also have resource implications, including costs for laboratory consumables, transport logistics, staff training and supervision, and maintenance of digital systems, which need to be factored into programme planning for sustainability.

This study has strengths and limitations that should be acknowledged. The primary strength and novelty of this study lie in its real-world, programme-embedded design and cascade-based evaluation, rather than in establishing a parallel research mechanism to provide insights directly actionable for programme managers. Second, the use of a structured cascade framework allowed systematic identification of stage-wise drop-offs. Third, universal inclusion of all eligible adults across three districts minimised selection bias and reflects heterogeneous district contexts. Several limitations warrant consideration. Reliance on routinely collected programme data may have introduced documentation bias and misclassification, with missing Ni-*kshay* entries potentially reflecting recording gaps rather than the absence of screening or action. Incomplete follow-up testing may have underestimated the true AT-DILI burden. As this was a single-arm descriptive feasibility study without a comparator, causal inference was limited, and no advanced analyses, such as multivariable, cluster-adjusted, or sensitivity analyses, were conducted, as the study was not designed for those purposes. The absence of qualitative and district-level health-system data limited exploration of inter-district variability. In addition, the study did not assess downstream clinical outcomes or cost-effectiveness, and the resource implications of wider scale-up remain uncertain.

This study has key policy implications and emerging recommendations. NTEP emphasises reducing TB-related mortality, preventing catastrophic costs, and strengthening patient-centric, differentiated care. While substantial gains have been made in case detection and treatment coverage, drug safety monitoring remains comparatively under-institutionalised. Therefore, it is prudent to reposition pharmacovigilance as a core quality indicator by incorporating it into routine quarterly reviews. Steps to integrate with the National Pharmacovigilance Programme should be taken. Incorporating follow-up LFT completion among persons at high-risk as a monitored programme metric within Ni*-kshay* dashboards would enhance accountability and align with the goal of reducing preventable early TB deaths. District-level heterogeneity observed in cascade performance suggests that supportive supervision and periodic performance feedback are critical. The district TB officers should monitor and evaluate AT-DILI screening cascade–related indicators in their periodic review. Further, differentiation of care must extend beyond disease severity to include treatment-related vulnerability. The experience from the TN-KET triage framework provides an opportunity to formally integrate AT-DILI risk stratification into standardised protocols. Recommending at least one follow-up LFT within 30 days for predefined high-risk groups could be operationalised within existing systems, although resource and implementation constraints needs consideration. Digital strengthening is essential to improve follow-up and documentation. Automated Ni-*kshay* alerts for pending or abnormal LFTs, integration of laboratory reporting with TB dashboards, and stronger referral-tracking mechanisms could enhance timeliness and ensure appropriate clinical action following AT-DILI diagnosis. Future research should explore predictors of cascade completion.

## CONCLUSION

Structured AT-DILI screening was partially feasible within routine NTEP workflows. While risk identification and baseline testing were achievable, important gaps remained in follow-up testing and documentation, and conclusions should be carefully drawn in the presence of inter-district variations and definitions of risk factors. Strengthening digital tracking, supervision, and accountability for referrals is essential to institutionalise proactive pharmacovigilance within TB care.

## Supplementary Material
