# Supplementary material for: Feasibility of tuberculosis drug–induced liver injury screening under programme settings in Southern India
Source: Public Health Action. 2026 Aug 24;16(3):156–61. doi: 10.5588/pha.26.0022 (PMC13502724; doi:10.5588/pha.26.0022)
Supplement: Supplementary file 1 [file pha26-0022_supplementarydata1.pdf]

Supplementary

Table 1. Detailed operational definitions of cascade indicators used for assessing the feasibility of AT-DILI screening among those at risk for AT-DILI among adults with TB started on 4Fixed dose combination ATT between July 2025 and December 2025, in 3 districts of Tamil Nadu.

Objectives	Data source	Methods	Outcome definition
**1. To estimate the implementation outcomes of the ATDILI cascade intervention in terms of:**			
**1a. Proportion of at-risk adults identified and documented in the Nikshay portal**	Ni-*kshay* records	Baseline extraction of all new adult with TB initiated on 4FDC.<br>A checklist of predefined ATDILI risk factors was applied.<br>Documentation in Nikshay and treatment cards were reviewed.	Proportion = (Number of patients with ≥1 risk factor)/ (Total adults with TB initiated during study period)
**1b. Proportion of at-risk adults who underwent AT-DILI screening within the first month**	Ni-*kshay* records	Cross-linking at-risk adults with LFT test dates. LFT considered valid if performed within 15-30 days of treatment initiation.	Proportion = (At-risk adults with documented LFT within 15-30 days)/ (Total at-risk patients)
**1c. Proportion of adults with AT-DILI linked to appropriate clinical management**	Ni-*kshay* records	Adults with abnormal LFTs (as per defined thresholds) were flagged. Subsequent documentation of treatment modification, referral, or specialist consultation were reviewed.	Proportion = (Adults with AT-DILI with documented action)/ (Total confirmed adults with AT-DILI)
**2. To estimate the service outcomes of the intervention in terms of:**			
**2a. Incidence of AT-DILI within the first month**	Lab reports documented in Nikshay	Based on AT-DILI criteria	Incidence: Number of adults with AT-DILI/Number of people at risk.
**2b. Median time from treatment initiation to LFT among high-risk adults**	Lab reports documented in Nikshay	Time difference between date of treatment initiation and receipt of the lab reports.	Time in days
**2c. Median time from abnormal LFT to initiation of clinical action**	Lab reports documented in Nikshay	Time difference between date receipt of the lab reports and change of clinical protocol.	Time in days

Table 2. Operational Cascade performance of the AT-DILI screening within 30 days of treatment initiation among adults with TB cohort initiated on ATT between July 2025 and December 2025, in 3 districts of Tamil Nadu. (N =2258)

**Stages-wise Indicators**	**Overall n(%)**	**District 1 n(%)**	**District 2 n(%)**	**District 3 n(%)**
**Stage 1: AT-DILI Risk Identification**				
Total registered on Ni-*kshay* & initiated on 4FDC	2258	459	667	1132
Date of ATT initiation available	2252(99.7)	453(98.7)	667(100)	1132(100)
Risk factors documented	2258(100)	459(100)	667(100)	1132(100)
Identified as AT-DILI at-risk	1180(52.25)	277(60.3)	438(65.7)	465(41.1)
Among those at risk (N=1180)				
Baseline LFT available	840(71.2)	273(98.5)	418(95.4)	149(32.1)
Dates of baseline LFT available	840(71.2)	273(98.5)	418(95.4)	149(32.1)
**Stage 2: Screening for AT-DILI among at-risk**				
Follow-up LFT	637(53.9)	193(69.7)	406(92.7)	38(8.2)
Date of Follow-up LFT available	629(98.7)	189(97.9)	402(99.0)	38(100)
Abnormal LFT detected at follow up				
Serum Bilirubin	13(2.0)	5(2.6)	4(1)	4(10.5)
AST	7(1.1)	0	3(0.7)	4(10.5)
ALT	50(7.8)	35(18.1)	11(2.7)	4(10.5)
**Stage 3: Linkage & Clinical Action**				
Referral documented among abnormal LFT	58(100)	35(100)	16(100)	7(100)

AT-DILI confirmed among AT-DILI at risk	18(1.5)	7(2.5)	5(1.1)	6(1.3)
Incidence proportion among at-risk (95% CI)	1.5(0.83-2.22)	2.53(0.68-4.38)	1.14(0.15-2.14)	1.29(0.26-2.32)

FDC -fixed dose combination, AT-DILI-anti-tuberculosis drug induced liver injury, AST-aspartate aminotransferase, ALT-alanine aminotransferase

Table 3. Operational definitions of the domains used for assessing the feasibility of AT-DILI screening among persons at risk for AT-DILI started on 4FDC between July 2025 and December 2025, in 3 districts of Tamil Nadu

**Indicator**	**Operational definition**
Coverage	Documentation of AT-DILI risk assessment at treatment initiation among eligible patients. Coverage ≥80% was considered acceptable for feasibility under routine program conditions.
Adherence	Completion of intended cascade steps among eligible individuals at each stage. Adherence ≥80% was considered acceptable for feasibility under routine program conditions.
Timeliness	Completion of follow-up LFT within 15–30 days of treatment initiation and initiation of clinical action following abnormal LFT results.
Predefined AT-DILI risk criteria	Age ≥60 years, severe undernutrition (BMI ≤16 kg/m²), alcohol use, tobacco consumption, HIV infection or concurrent antiretroviral therapy, pregnancy or early postpartum status, or triage positivity under TNKET risk stratification.
AT-DILI	Presence of any one of the following: (i) AST or ALT >5× upper limit of normal (ULN); (ii) total bilirubin >2 mg/dL with AST/ALT or ALP >2× ULN; or (iii) AST/ALT >3× ULN with compatible symptoms.

ALP = alkaline phosphatase, ALT = alanine aminotransferase, AST = aspartate aminotransferase, LFT = liver function test, ULN = upper limit of normal, TNKET= Tamilnadu kaasanoi erapilla thittam.
